# Fault-Tolerant Electro-Responsive Surfaces for Dynamic Micropattern Molds and Tunable Optics

**DOI:** 10.1038/s41598-017-12899-y

**Published:** 2017-10-02

**Authors:** I-Ting Lin, Tiesheng Wang, Fenghua Zhang, Stoyan K. Smoukov

**Affiliations:** 1 Department of Materials Science and Metallurgy, University of Cambridge, Cambridge, CB3 0FS United Kingdom; 2 Centre for Composite Materials and Structures, Harbin Institute of Technology, Harbin, 150080 People’s Republic of China; 3 School of Engineering and Materials Science, Queen Mary University of London, London, E1 4NS United Kingdom; 4 Department of Chemical and Pharmaceutical Engineering, Sofia University, Sofia, 1164 Bulgaria

## Abstract

Electrically deformable surfaces based on dielectric elastomers have recently demonstrated controllable microscale roughness, ease of operation, fast response, and possibilities for programmable control. Potential applications include marine anti-biofouling, dynamic pattern generation, and voltage-controlled smart windows. Most of these systems, however, exhibit limited durability due to irreversible dielectric breakdown. Lowering device voltage to avoid this issue is hindered by an inadequate understanding of the electrically-induced wrinkling deformation as a function of the deformable elastic film thickness. Here we report responsive surfaces that overcome these shortcomings: we achieve fault-tolerant behavior based on the ability to self-insulate breakdown faults, and we enhance fundamental understanding of the system by quantifying the critical field necessary to induce wrinkles in films of different thickness and comparing to analytical models. We also observe new capabilities of these responsive surfaces, such as field amplification near local breakdown sites, which enable actuation and wrinkle pattern formation at lower applied voltages. We demonstrate the wide applicability of our responsive, fault-tolerant films by using our system for adjustable transparency films, tunable diffraction gratings, and a dynamic surface template/factory from which various static micropatterns can be molded on demand.

## Introduction

Dynamic control of surface topography and roughness is highly desired for its potential to accomplish what is difficult for traditional static surfaces to achieve, such as adjustable wettability^1^, smart adhesion^2^, antifouling abilities^3^, tunable light diffraction^4^, e-skin and stretchable devices^5,6^. Depending on the material’s mechanical properties, such surfaces can show a variety of topographies during deformation, including wrinkling (or buckling), formation of localized ridges, period-doubling, creasing, and delamination^7,8^. Several methods have been used to induce such surface deformation, such as in-plane mechanical compression^9–11^, relaxation after depositing thin films on pre-stretched substrates^12–17^, thermal expansion mismatch after cooling thin films deposited on hot substrates^18–22^, ion irradiation^23,24^, differential swelling of film layers^2,25–29^, and electric-field-induced deformation^30–32^.

Among these various approaches, electric-field-induced deformation is particularly attractive due to its ability to reversibly actuate at high frequency and its potential for miniaturization and programmable control^33^. This type of deformation relies on the concept of voltage-induced electromechanical instability^34–39^, a phenomenon frequently observed in dielectric elastomer actuators (DEAs)^40–45^. Wang and Zhao, for example, have produced creased/cratered/wrinkled structures by immobilizing a DEA on a rigid substrate^32,46^. This DEA-like system is composed of four layers from top to bottom: a conductive liquid (e.g. NaCl solution) as a top electrode, an elastic layer, a rigid layer, and a bottom electrode. When a high voltage is applied across the two electrodes, the elastic layer is squeezed and thus tends to expand laterally due to its incompressibility. Meanwhile, the rigid layer not only provides a higher breakdown strength, but it also confines the elastic layer and blocks its expansion. The resulting lateral compressive stresses induce instabilities which deform the surface structure. Designs such as these pave the way for more practical applications such as responsive anti-biofouling surfaces in marine environments^3^. van den Ende *et al*. have shown further application of these DEA-based systems by creating smart windows and mirrors based on wrinkling-induced light diffusion^31^.

In order to incorporate these DEAs into real-world applications, however, it will be necessary to address the issues with dielectric breakdown which limit the durability of these systems. Local inhomogeneity, defects, and large deformations during actuation in DEA-like systems may create some locally thinner areas (e.g. depressions of creases and wrinkles) which, at constant surface potential, create high local fields. These high fields drive the film to become even thinner in these locations, resulting in a positive feedback loop which leads to eventual dielectric breakdown and thus short circuiting^40^. This breakdown vulnerability is catastrophic and makes many DEA devices short-lived. Efforts to circumvent this breakdown issue by inducing wrinkling at lower voltages have been hindered by a limited understanding of the factors which affect the critical voltage for wrinkling, such as device thickness.

**Figure 1:**
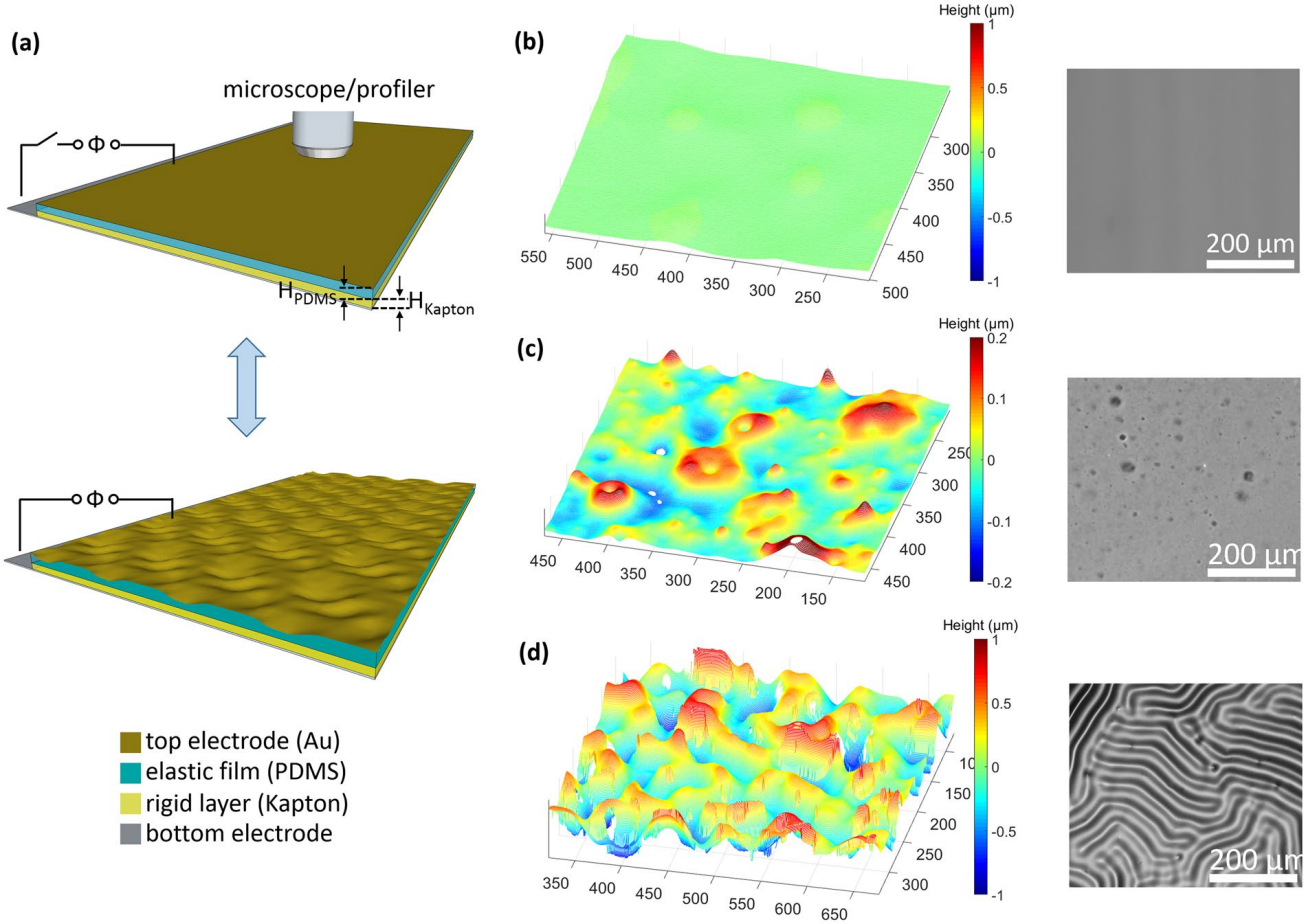
(**a**) Concept of DEA-like responsive surfaces. When voltage is applied, the PDMS layer and coated gold film deform due to the electric field (oriented normal to the surface). (**b**) Flat surface at 0–3 kV, as observed by optical profiler (left) and optical microscope (right). (**c**) Formation of craters at 4 kV. (**d**) Formation of wrinkles at 5 kV. H_PDMS_ is 22 µm and H_Kapton_ is 50 µm for each of the samples shown in (**b**–**d**).

Here we report the fabrication and characterization of a fault-tolerant electrically responsive surface (Fig. 1a) that addresses these issues and introduces novel capabilities for a variety of applications. The fault-tolerance is due to self-insulation of localized breakdown, and presence of multiple defects does not prevent the actuation. Furthermore, targeted breakdown can be used to create patterned morphologies under lower voltages. We contribute to the fundamental understanding of these DEA-like systems by investigating how the critical voltage/field necessary to induce surface wrinkles varies with the elastic film thickness (from 9 µm to 83 µm). Finally, we demonstrate potential applications in tunable transparency films, dynamic diffraction gratings, and dynamic surface templates using commercially available materials for the rigid layer (Kapton tape), elastic layer (PDMS), and integrated top electrode (Au).

## Results and Discussion

### Dynamic Topography at Different Voltages: From Cratering to Wrinkling

One of the most common approaches to achieve large topographical changes is to wrinkle the surfaces of bilayer films, in which the top film is much stiffer than the bottom elastic layer. In this system, lateral (in-plane) compressive stress above a critical value will cause strain mismatch between the two layers and induce wrinkling^9^. Wrinkling of a surface can also be induced by applying an out-of-plane force, normal to an elastomeric coating^47^. This model is natural to apply to our DEA-like system, as the applied electric field generates an out-of-plane force. To make a device working with this out-of-plane model, we fabricate it with four layers from the top to the bottom (Fig. 1a): a thin gold film as the top electrode, an elastic dielectric layer made of polydimethylsiloxane (PDMS), a rigid layer (Kapton film), and a gold-coated glass slide as the bottom electrode. The top electrode can buckle with the compliance of the PDMS elastic dielectric layer underneath. PDMS is not directly coated on the bottom electrode since its dielectric strength (21 kV/mm) is much lower than the critical electric field required to induce wrinkling. Instead, PDMS is coated on a 50 µm thick Kapton film with dielectric strength of 197 kV/mm to prevent breakdown before the applied voltage reaches the critical wrinkling value.

When applying voltage, reversible wrinkles can be observed on the surface of the gold top electrode through both an optical profiler and optical microscope, as depicted in Fig. 1a. While the optical profiler shows a detailed height map of the surface profile, the optical microscope gives a quick two-dimensional view of the pattern structure. Figure 1b–d show images of the flat state (0 to 3 kV), and actuated states at 4 kV and 5 kV. It can be seen in Fig. 1b that when the voltage is below 3 kV, the surface is relatively smooth, since the force is not sufficient to deform the surface. When the voltage is 4 kV (Fig. 1c), some craters are observed on the surface. These craters can be attributed to defects due to voids, partial curing, or the intrinsic inhomogeneity of polymer, which may concentrate the local field or stress and make some small regions become thinner when the voltage is applied. Since it has been reported that charges tend to flow to thinner areas of dielectric elastomer actuator^48^, this process leads to charge localization and forms craters before the whole film is wrinkled. At 5 kV (Fig. 1d), the induced out-of-plane force reaches the critical value required to create wrinkles on the surface. We thus demonstrate that different surface patterns can be generated, namely craters at 4 kV and wrinkles at 5 kV, simply by applying different voltages to generate different amounts of out-of-plane force.

**Figure 2:**
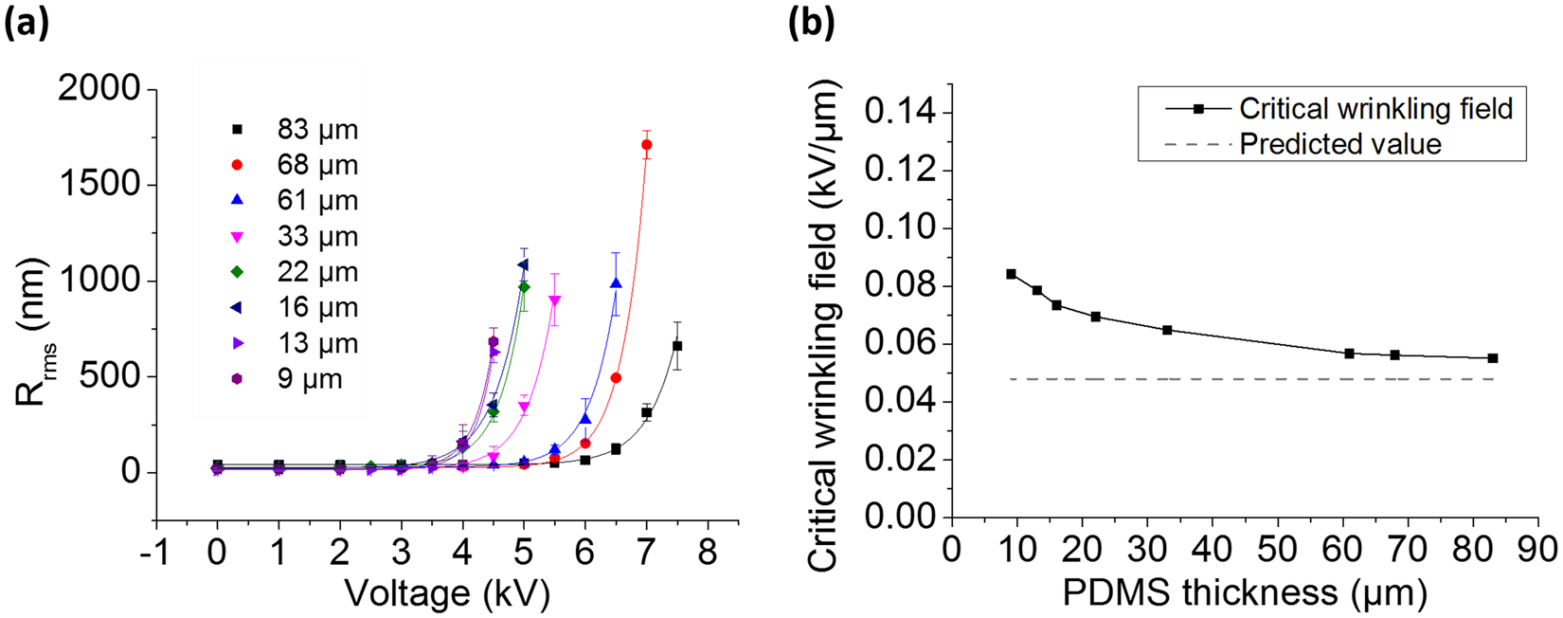
(**a**) Surface roughness at different applied voltages for PDMS thicknesses ranging from 9 to 83 µm. The critical voltage is determined by the empirical function R_rms_ = R_0_ + 120 × exp (*k* (Φ−Φ_c_)) nm, where R_0_ is the roughness at Φ = 0. (**b**) Critical electric field for wrinkling of Au on different thicknesses of PDMS. As the PDMS thickness decreases, the curve deviates from the theoretical value (0.048 kV/µm), which assumes the top gold film to be infinitesimally thin.

In addition to a detailed profile of the wrinkled structure, the optical profiler also measures surface roughness of larger areas. Measuring roughness (see Methods) as a function of applied voltage provides a facile means of determining the critical voltage and corresponding electric field that induces wrinkling. Figure 2a plots the root mean squared roughness, R_rms,_ of the surface at various applied voltages for different thicknesses of PDMS. The critical wrinkling voltage, Ф_c_, for each thickness of PDMS is determined by fitting the data with an empirical exponential growth function: R_rms_ = R_0_ + 120 × exp (*k* (Φ−Φ_c_)) nm, where R_0_ is the roughness when the applied voltage is zero and *k* is the exponential growth constant.

To further relate the critical voltage to the critical field, the thickness of the dielectric layer needs to be incorporated in the above equation. Since a Kapton film is added underneath the PDMS to increase the breakdown voltage, the effective thickness of dielectric layer should be considered and the electric field in the PDMS film can be expressed as^32^:

1 $${E}_{PDMS}=\frac{\Phi }{{H}_{PDMS}+{H}_{Kapton}\cdot {\varepsilon }_{PDMS}/{\varepsilon }_{Kapton}}$$

where Φ denotes the applied voltage, H is the thickness of each layer and ε is its corresponding electrical permittivity. Here ɛ_PDMS_/ε_Kapton_ equals to 2.65⋅ε_0_/3.5⋅ε_0_ = 0.76. The derived critical wrinkling fields for different thicknesses of PDMS are shown in Fig. 2b. This experimental data is in reasonable agreement with that of the out-of-plane force-induced wrinkling model developed by Huang^49^, which predicts a critical wrinkling field of 0.048 kV/µm (shown as the dashed line in Fig. 2b). In this model, the critical field to induce wrinkling in PDMS can be expressed as:

2 $${E}_{c}=\sqrt{\frac{{\xi }_{c}{Y}_{PDMS}}{{\varepsilon }_{PDMS}}}$$

where ${{\rm{Y}}}_{{PDMS}}$ is the Young’s modulus (83 kPa, see Method section) and ɛ is the dielectric constant (ɛ_PDMS_ = 2.65⋅ε_0_). ξ_c_ is a constant based on Poisson’s ratio. ξ_c_ = 2/3 when the Poisson’s ratio is 0.5 for PDMS^31^.

Note that our observed critical wrinkling field increases relative to the predicted value of equation (2) with decreasing PDMS film thickness. This discrepancy may be attributed to the fact that the model assumes the top electrode to be infinitesimally thin^49^, an assumption which can never be satisfied in reality. Thus, the measured critical field approaches the predicted value as the PDMS film thickness increases, when the top gold electrode is relatively thinner. This trend is consistent with a theoretical study of in-plane compression on similar systems which did not assume an infinitesimally thin top film^50^.

When designing a DEA-like device, a common strategy to reduce the working voltage is to decrease the film thickness so that the same field can be maintained at lower applied voltage. However, our trend in critical wrinkling field versus PDMS film thickness implies that decreasing PDMS thickness does not always lead to a proportional reduction of the working voltage. Further characterization on devices with very low PDMS thicknesses will be necessary to fully understand these systems.

Since we select commercially available materials (Kapton tape, PDMS, etc.) with low price for ease of fabrication, the working voltage shown here is relatively high (3 to 7 kV). We have demonstrated that using different materials for the elastic and rigid layers with the same design might reduce the voltage (Supplementary Fig. S2). For example, from equation (1) we know that an elastic layer with a low Young’s modulus and high dielectric constant can reduce the critical field for wrinkling, and from equation (2) the field can be amplified under the same applied voltage if choosing a material with thinner thickness or higher dielectric constant for the rigid layer.

### Fault-Tolerant Surface and Defect-Amplified Electric Field

Many real-world applications of these electrically-actuated responsive surfaces require large-scale deformations, such as strong light diffusion in tunable optics or rough texture change for dynamic camouflage. Achieving these large topographical changes typically requires high actuation voltages. These high voltages, however, can cause dielectric breakdown of the DEA system that eliminates its ability to act as a responsive surface^51,52^. Therefore, most DEA-based responsive surfaces are restricted to relatively low voltage ranges, limiting their ability to achieve significant levels of deformation. This issue motivates an investigation into potential mechanisms for (1) achieving fault-tolerant behavior, in which the surface can still actuate after some degree of dielectric breakdown, and (2) lowering the applied voltage levels necessary to achieve large-scale surface deformation.

**Figure 3:**
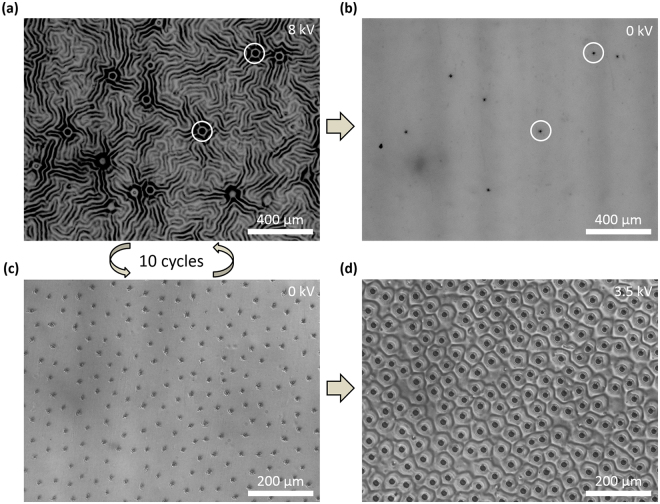
(**a**) Local breakdown on the surface during deformation. The white circles highlight areas of puncture. (**b**) The formed punctures after the surface is returned to its resting state (0 kV). (**c**) The surface at 0 kV after 10 actuation cycles. (**d**) Actuation on a punctured surface.

We explore the effects of dielectric breakdown on our system by applying voltages much higher than Φ_c_ (the critical voltage to induce wrinkling). For example, as we tuned the applied voltage from 0 to 8 kV across a 22 µm PDMS film, the wrinkles appear at Φ_c_ = 4.17 kV (green line in Fig. 2a) and create valleys on the surface. When the voltage is raised further, the surface roughness and wrinkle depth also increase, making the film at the bottom of these wrinkle valleys even thinner. For a constant applied voltage, locally thinner areas in the film experience much higher electric fields, and that is where breakdown occurs when the applied voltage is high enough. These high local electric fields across the film result in high leakage current flowing through the elastomer and cause Joule heating and higher local conductivity^53^, resulting in a positive feedback loop until the short circuit current is high enough to cause the breakdown and the burning of the conductive 16 nm Au material around the breakdown fault, leaving even the damaged dielectric insulated from further breakdown. Video SV1 in supporting information shows the formation of the local breakdown when the applied voltage reaches 8 kV, and Fig. 3a highlights the breakdown regions in red circles. The current generated from this process can be observed using the amperometer of the power supply, and it vanishes once the Au around the punctures has burned away, which leaves the electrodes disconnected. After removing the applied voltage, we observe punctures formed from these small burned regions on the surface (Fig. 3b). The number of these breakdown punctures saturates after several high voltage cycles, as shown in Fig. 3c. Note that because these regions of local breakdown are insulated from the rest of the surface, immediate large-scale breakdown of the device is prevented and the punctured film can still actuate for many times (Fig. 3d); similar self-clearing behavior has been observed in DEA artificial muscles under large deformation when single-walled carbon nanotubes are used as electrodes^54^. This fault-tolerant behavior in our DEA-like system prevents malfunction even under high voltage, which prolongs life of the system and simplifies the manufacturing process, since even slightly defective films can still actuate.

In addition to enabling fault-tolerant behavior, the local breakdown phenomena we observe in our DEA-like system can also be used to activate our surfaces at lower voltages in the presence of breakdown-induced punctures. Through optical profiler measurements, we observe that the voltage required to reach a roughness of about 900 nm has decreased from 5 kV to 3.5 kV, a reduction of 30%. We hypothesize that edge effects and charge accumulation around punctures in the film amplify local electric fields, as has been observed previously for capacitors^55,56^. This amplified field reduces the minimum voltage necessary to actuate the surface near the punctures. We thus present an unconventional method to decrease the need for high working voltages, which has always been a concern for DEA-like systems. As mentioned in the previous section (Fig. 2b), reducing the voltage by decreasing device thickness may also increase the minimum field necessary to induce wrinkling. The result here provides another strategy by locally enhancing the electric field near electrodes or puncture sites.

### Applications

#### Tunable Transparency Film

**Figure 4:**
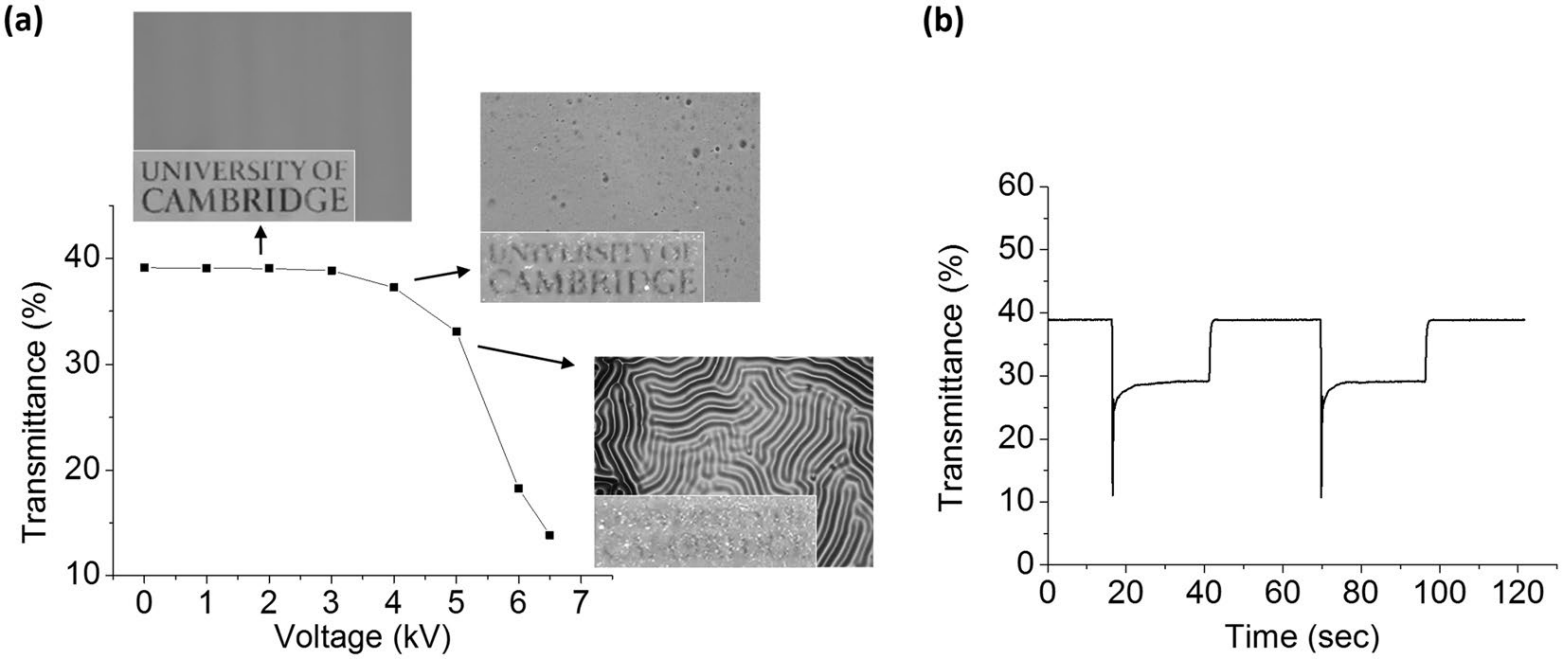
(**a**) Electro-dynamically tunable light diffusion for films at different voltages, and (**b**) kinetics and cycling stability of the transmittance response. Logo usage is approved by University of Cambridge.

One of the most promising applications of these DEA-like systems is in films or windows with tunable transparency^57^ (Video SV2 in supporting information). As the applied voltage is gradually increased and the surface topography changes, the transmittance through the film also changes due to optical diffusion. As shown in Fig. 4a, the transmittance drops slightly once craters appear on the surface, then further decreases near 5 kV when the wrinkles diffuse more light. When increasing the voltage to 6 kV, the amplitude of the wrinkles increases to yield even lower light transmittance. This tunable transparency phenomenon is reversible and stable for many cycles, as shown in Fig. 4b.

Note that the sharp drops in transmittance at 17 and 70 seconds in Fig. 4b are due to inductive voltage spikes which appear when switching on the power supply^58^. This voltage spikes come from inductive loads which can generate magnetic field when current flow through them at the moment the power supply is switched on/ off. The peak voltage induces large topographical changes, resulting in very low transmittance. The transmittance drops immediately from 39% to 11% and relaxes back to about 28% after a few seconds. However, the short-lived peak voltage does not cause dielectric breakdown in the system, as demonstrated by the fact that the device can work reproducibly for several cycles (Fig. 4b). It has been reported that working above the breakdown voltage for a short time period will not irreversibly damage DEA systems^59^.

#### Dynamic Diffraction Grating

**Figure 5:**
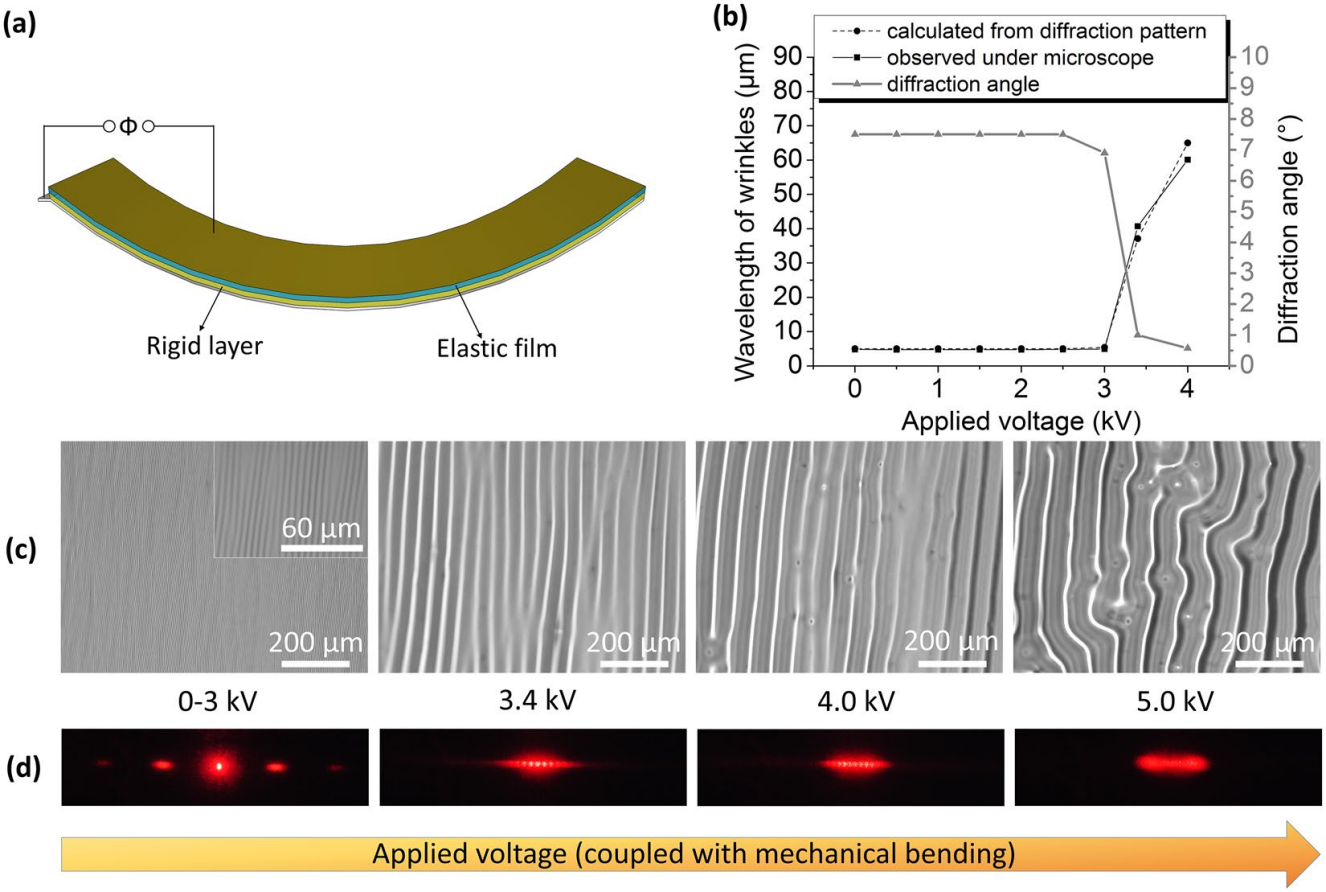
Applications in tunable diffraction gratings. (**a**) Setup for mechanically inducing wrinkles, the spacing of which can be electrically tuned. (**b**) Measured diffraction angle, calculated d value, and observed wrinkle spacing as a function of applied voltage. (**c**) Images of directional wrinkles at different voltages, and (**d**) the corresponding diffraction patterns.

Besides smart windows, this responsive surface can also achieve dynamic diffraction grating for applications such as wavelength selection, masks for photolithography, or astronomy and space instrument^60^. This application requires regularly-spaced, unidirectional wrinkles, rather than the randomly-oriented patterns described previously. We achieve these patterns by coupling the voltage-induced wrinkling process with mechanically-induced wrinkles, formed upon bending the structure (Fig. 5a). This process yields two sets of wrinkles: primary wrinkles, appearing at a certain critical stress, and secondary wrinkles, induced under higher stress^10,61^. In this experiment we induce the primary wrinkles by mechanical bending the film, inducing a stress lower than where secondary wrinkles would appear. It is not well known what the critical stress is in our system that induces the secondary wrinkles, but by using electrical voltage, we could alter the total stress applied and cross into the secondary wrinkle generation, which results in the ability to tune the wavelength of existing wrinkles for light grating. The wrinkle spacing can be characterized optically by diffraction:

3 $$n\lambda =d\cdot \,\sin \,\theta $$

where *d* is the separation of grating elements (spacing of wrinkles here), and can be calculated from the light wavelength λ (650 nm) and the angle between successive diffraction orders θ, and n is a non-zero integer.

The diffraction angle and calculated *d*, shown in Fig. 5b, are consistent with the wrinkle spacings directly observed by microscope. Figure 5c shows optical images of directional wrinkles under different applied voltages, and Fig. 5d shows the corresponding diffraction pattern. When no voltage is applied, primary wrinkles form due to mechanical bending alone. As the figure shows, primary wrinkles with a spacing of 5 µm can be clearly observed, whereas secondary wrinkles with larger spacing are relatively shallow and insignificant, so the formed diffraction pattern is mainly due to the primary wrinkles. When a voltage of 3.4 kV is applied, the additional electric force facilitates the formation of secondary wrinkles, which emerge and propagate from the existing shallow secondary wrinkles induced from mechanical bending. The larger spacing (41 µm) of these secondary wrinkles yields a correspondingly smaller diffraction pattern spacing. When the voltage increases to 4 kV, the spacing of the wrinkles further increases to 60 µm. This value is similar to the spacing achieved using pure electric compression without mechanical bending (Fig. 1d), indicating that electrically-induced wrinkling starts to dominate the overall wrinkling mechanism. At even higher voltages such as 5 kV, the isotropic electrically-induced wrinkling dominates the anisotropic mechanically-induced compression, such that the wrinkles tend to lose unidirectionality and the diffraction pattern blurs.

#### Dynamic Factory for Fabricating Permanent Patterns

**Figure 6:**
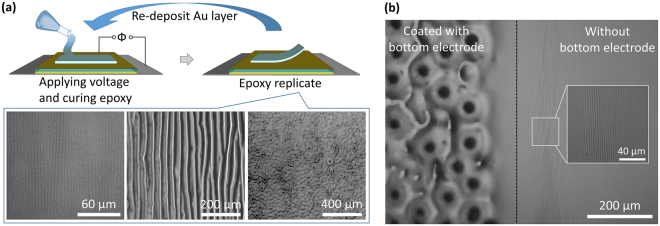
(**a**) Process of dynamic micropattern fabrication, with images of replicated patterns created from the reusable DEA-like template (from left to right: 0 kV with mechanical bending, 3.4 kV with mechanical bending, and 5.5 kV without mechanical bending). (**b**) Demonstration of the fabrication of different patterns on the same film.

The applicability of this DEA-like system can be extended even further by replicating and preserving our voltage-induced surface patterns, which we accomplish using molding. By pouring uncured epoxy on the responsive surface and applying voltage while the epoxy cures, we can obtain a copy of the surface craters or wrinkles, as depicted in Fig. 6. Removing the applied voltage and peeling off the epoxy results in a replicate of the wrinkled structure. In this process, the gold layer comprising the top electrode is transferred to the epoxy without damaging the underlying PDMS. Thus, after sputtering gold on the PDMS again, the immobilized DEA system can be reused to mold another pattern. We demonstrate several patterns made from the reusable pattern factory in Fig. 6a. This pattern replication operation is carried out at room temperature and requires only 30 minutes for epoxy curing and 5 minutes for re-deposition of the gold top electrode, enabling easy and fast fabrication for different replicated patterns.

Furthermore, this technique can even be used to create multiple patterns on the same film, which is an advantage over traditional molding processes based on mechanically-induced wrinkles. This complex patterning is achieved by altering the design of the bottom electrode, as exemplified in Fig. 6b, in which only the left half of the film is supported by the gold bottom electrode underneath the Kapton tape. The sample is first mechanically bent (as in Fig. 5a) to obtain unidirectional wrinkles on the entirety of the film. Then, we apply 7 kV to create punctures on the left half of the surface as a pre-treatment and subsequently remove the voltage. When applying voltage again (4 kV), the portion with the gold bottom electrode will be affected by the field and show wrinkles around the punctures, while the other part will only maintain the small-scale, mechanically-induced wrinkles.

## Conclusions

In summary, we demonstrate a simple method for fabricating fault-tolerant electrically-responsive surfaces based on commercially-available materials and standard metal evaporation technology. The DEA-like system can produce different surface topographies upon application of voltage and remains functional due to self-insulation even after multiple high voltage breakdown punctures. Targeted breakdown punctures can be used for amplification of local electric fields for actuation at lower working voltages. We find that the critical wrinkling field increases with decreasing thickness of the elastic layer, in quantitative agreement with the out-of-plane wrinkling model, with potential to assist in different device designs. Optical applications would benefit from using a high transmittance material as the rigid film to achieve a highly transparent off-state. Lowering the Young’s modulus of the elastic layer, decreasing the thickness of the rigid layer, and increasing the dielectric constant of both, would decrease the working voltage and enhance the performance of the demonstrated (changeable transparency films, dynamic light-grating filter, and a template/factory for static surface patterns) and other applications this versatile, low-cost technology.

## Methods

### Experimental Setup

A schematic of our DEA-like system is depicted in Fig. 1a. The bottom electrode was formed by sputter-coating (K550 sputter coater, Emitech, Inc.) a 16 nm thick layer of gold onto a glass slide. As the rigid layer for the device, a polyimide film (50 µm thick Kapton tape, DuPont, Young’s modulus 2.5 GPa) was adhered to the bottom electrode. The elastic dielectric layer of the devices was formed using PDMS (Sylgard 184, Dow Corning): a solution with 3 wt.% crosslinker in the matrix base was diluted in toluene (90%) and stirred for 5 hours to achieve homogeneity. The solution was then spin-coated (Laurell WS-650MZ-23NPP) on the Kapton film at speeds ranging from 500 to 5000 rpm to achieve layers of different thicknesses. The samples were then cured in an oven at 45 °C overnight to form solidified elastomer films with a Young’s modulus of approximately 83 kPa. A thin gold film (16 nm, Young’s modulus 36 GPa) was subsequently sputter-coated on the top of the PDMS to create the top electrode. For mechanically bent films, the bottom electrode was made flexible by coating the gold layer on an overhead projector transparency film (Nobo ACCO Brands). The films shown in Figs 5 and 6b are bent to a curvature of −0.38 cm^−1^. Electrically-induced deformation of the film surfaces was achieved using a HV Power Supply (Gamma High Voltage Research). For micropattern fabrication, an epoxy resin base (reaction product: bisphenol-a-(epichlorhydrin), epoxy resin with average molecular weight >700, butyl 2,3-epoxypropyl ether) and hardener (polyamide resin) were used and cured at room temperature for 30 minutes.

### Measurement

The final film thicknesses were measured by a Dektak 6 M Stylus Profiler. An Olympus Reflected Fluorescence system BX51 with a charge-coupled device (CCD) (INFINITY3-3URM from Lumenera Corporation) was used to observe the surface patterns by optical microscope. 3D mapping profiles and surface roughness were measured using a Zygo Newview Interferometric Profilometer. The surface roughness, R_rms_, is defined as the root mean square of the height differences between each point on the surface and the mean height. For measurements of Young’s modulus, mechanical testing was done with a Hounsfield Low Load Electric Screw Machine (5 kN) and a MTS Nanoindenter XP.

### Data Availability Statement

All the details and parameters of the experiments are described in Methods Section and Supplementary Information.

## Electronic supplementary material

Supplementary Information

Video SV1

Video SV2

Video SV3
