# Supplementary material for: Fault-Tolerant Electro-Responsive Surfaces for Dynamic Micropattern Molds and Tunable Optics
Source: Sci Rep. 2017 Oct 2;7:12481. doi: 10.1038/s41598-017-12899-y (PMC5624962; doi:10.1038/s41598-017-12899-y)
Supplement: Supplementary file 1 — Supplementary Information [file 41598_2017_12899_MOESM1_ESM.pdf]

Supplementary Information

Fault-Tolerant Electro-Responsive Surfaces for Dynamic Micropattern Molds and Tunable Optics

I-Ting Lin[1], Tiesheng Wang[1], Fenghua Zhang[1,2], and Stoyan K. Smoukov[1,3,4,*]

[1]Department of Materials Science and Metallurgy, University of Cambridge, Cambridge CB3 0FS, United Kingdom
[2]Centre for Composite Materials and Structures, Harbin Institute of Technology, Harbin 150080, People's Republic of China
[3]School of Engineering and Materials Science, Queen Mary University of London, London E1 4NS, United Kingdom
[4]Department of Chemical and Pharmaceutical Engineering, Sofia University, Sofia 1164, Bulgaria
*sks46@cam.ac.uk
s.smoukov@qmul.ac.uk

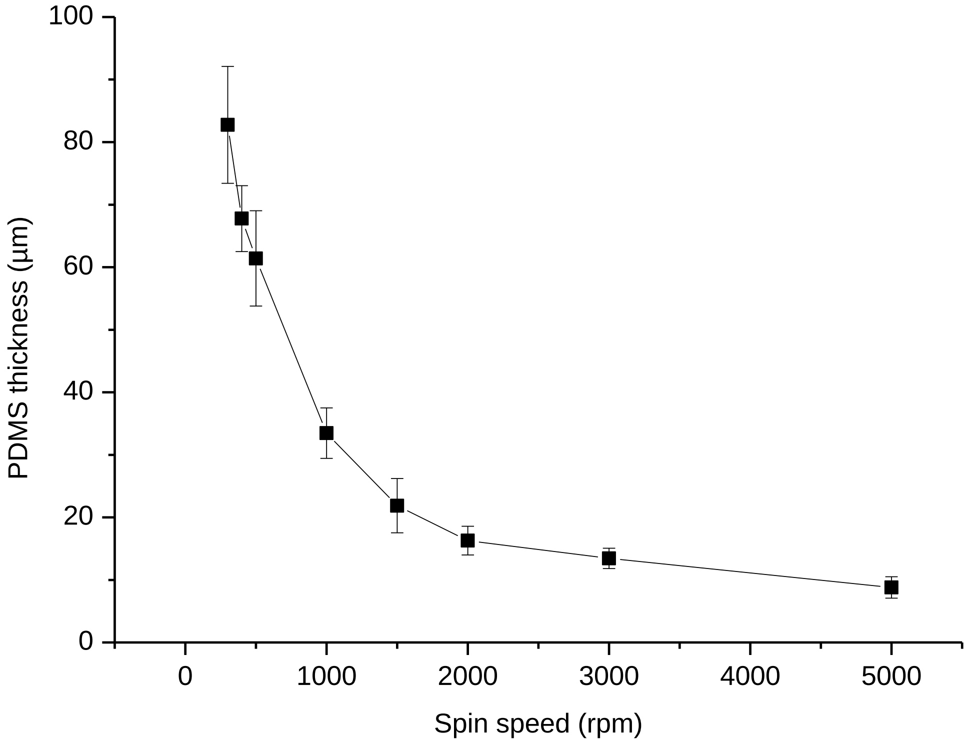

Figure S1. Different thicknesses of Sylgard 184 PDMS film achieved by spin coating at different speeds (3% crosslinker to 97% base ratio, and then diluted 9:1 (w/w) in toluene).

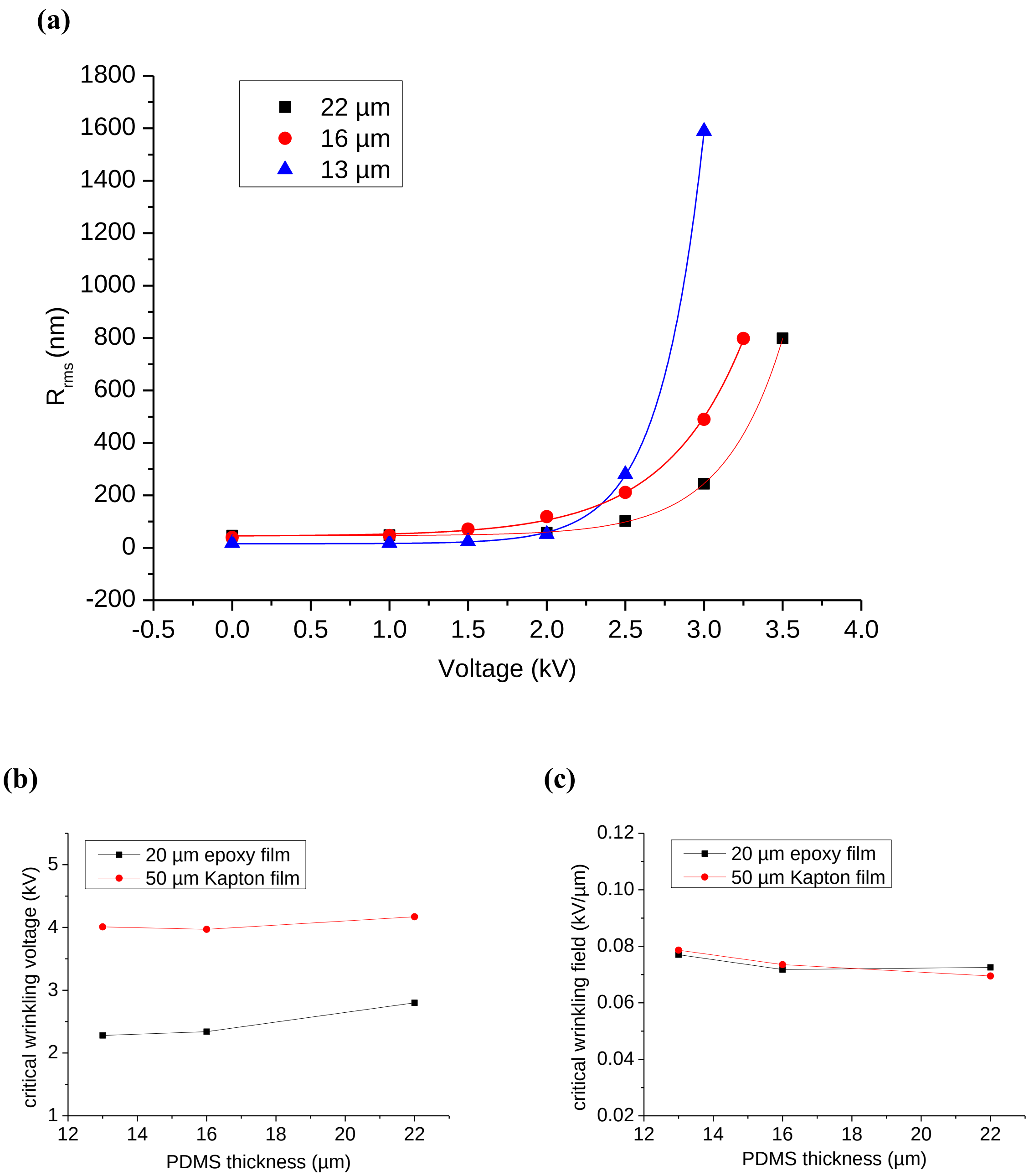

Figure S2. (a) Surface roughness at different voltages for 13, 16, and 22 µm thick PDMS coated on SU-8 epoxy as the rigid layer. (b) Critical wrinkling voltage for epoxy- based and Kapton-based system. (c) Critical wrinkling fields in PDMS layer to deform the surface for both systems are similar. Note that the dielectric constant is 3.5 for Kapton and 3.2 for SU-8 epoxy.

Video SV1. Video of the local breakdown process (PDMS thickness = 22 μm, and Kapton film thickness = 50 μm. Applied voltage is 8 kV).

Video SV2. Video for tunable transparency (PDMS thickness = 22 μm, Kapton film thickness = 50 μm, and electrodes are 16 nm thick gold layer. Applied voltage is ramped up from 0 to 6 kV). Logo usage is approved by University of Cambridge.

Video SV3. Video for dynamic diffraction grating (conditions shown in main text).
